# Sex differences in schizophrenia: symptomatology, treatment efficacy and adverse effects

**DOI:** 10.3389/fpsyt.2025.1594334

**Published:** 2025-06-16

**Authors:** Ivi Moniem, Vasilios Kafetzopoulos

**Affiliations:** ^1^ Department of Oncology, Nicosia General Hospital, Nicosia, Cyprus; ^2^ Department of Psychiatry, Medical School, University of Cyprus, Nicosia, Cyprus

**Keywords:** sex differences, schizophrenia, symptomatology, adverse effects, personalized medicine

## Abstract

**Introduction:**

This systematic review highlights that schizophrenia (SZ) manifests significant sex differences across neurobiological, clinical, treatment response, and adverse effect domains, underscoring the need for sex-specific considerations in both research and clinical practice.

**Methods:**

A systematic search was conducted following PRISMA guidelines, primarily through Pubmed, PsycINFO, Web of Science, and the Cochrane Library. Eligible studies on sex differences in patients with schizophrenia were included. Main search keywords were *schizophrenia, sex differences, sex, gender, neurobiology, symptomatology, epidemiology, treatment response, adverse effects*.

**Results:**

While lifetime prevalence is similar between men and women, the disorder’s trajectory diverges. Men typically experience illness onset approximately 3–5 years earlier than women, with more severe negative symptoms, worse social functioning, and higher rates of comorbid substance use disorders. By contrast, women often have later onset—including a secondary mid-life peak likely linked to declining estrogen—and tend to present with more affective symptoms. Neurobiologically, men with SZ exhibit more extensive structural brain abnormalities and cognitive impairment (especially in memory), whereas women benefit from a degree of neuroprotection possibly mediated by estrogen and distinct gene expression patterns. Hormonal influences appear pivotal, with estrogen’s neuroprotective effects potentially delaying onset and mitigating symptom severity in women, and low testosterone levels correlating with more pronounced negative symptoms in men. Treatment response also varies by sex: women with SZ generally respond to antipsychotics at lower doses with better clinical improvement and fewer relapses, whereas men often require higher doses due to faster drug metabolism and also typically face higher relapse risks. Notably, the treatment advantage of women diminishes after menopause. Adverse effect profiles differ as well: women with SZ are more prone to side effects such as antipsychotic-induced hyperprolactinemia, weight gain, and metabolic or cardiovascular complications, while men tend to experience more neurological side effects while also exhibiting lower treatment adherence.

**Discussion:**

These multidimensional sex differences underscore that all aspects of schizophrenia—from pathophysiology and presentation to therapy and side effect management—must be viewed through a sex-specific lens. Tailoring interventions to the needs of men and women patients is essential for optimizing outcomes and advancing personalized care.

## Introduction

Schizophrenia (SZ) is a severe psychiatric disorder with a complex etiology and heterogeneous clinical presentation. A consistent finding in the epidemiology of schizophrenia is that its incidence and course differ between men and women. Men have a higher incidence of schizophrenia (man-to-woman ratio ~1.4:1) and tend to develop the illness earlier in life (1). By contrast, women on average experience a later onset – often several years after the typical peak in men – and frequently display better premorbid social functioning (2, 3). Clinically, it can be argued that men more frequently present with prominent negative symptoms and poorer social adjustment, whereas women tend to exhibit more affective symptoms (e.g., depression) and acute psychotic features (4, 5). These differences may contribute to divergent outcomes: some studies report that women achieve higher initial remission rates and milder early-course illness severity (potentially due to estrogen’s neuroprotective effects) (6). Indeed, estrogen is hypothesized to delay illness onset and reduce symptom severity in women until menopause (7). Conversely, men often have a more chronic course with worse long-term functional outcomes, partly attributable to poorer premorbid development and greater substance use comorbidity (8, 9).

Growing recognition of these sex differences has spurred a robust body of literature exploring biological and psychosocial distinctions between men and women with schizophrenia. Evidence has emerged that sex-based factors can influence nearly every aspect of the disorder – from neurodevelopmental pathways and brain morphology to clinical symptomatology, treatment response, and side-effect profiles (9–11). For example, sex-specific genes and hormones may differentially impact brain structure and function, contributing to variations in disease risk and presentation (12). Likewise, gender-related social roles and behaviors (e.g., substance use patterns) can modify the clinical course (13). These differences have practical implications: women and men may respond differently to antipsychotic treatments and experience distinct adverse effect burdens (14, 15). Yet, historically, many treatment guidelines have not accounted for sex, and clinical trials often included predominantly men samples (16–18). There is a pressing need to synthesize current evidence on sex differences in schizophrenia to inform more personalized, gender-sensitive care (19–21).

## Methods

**Figure 1:**
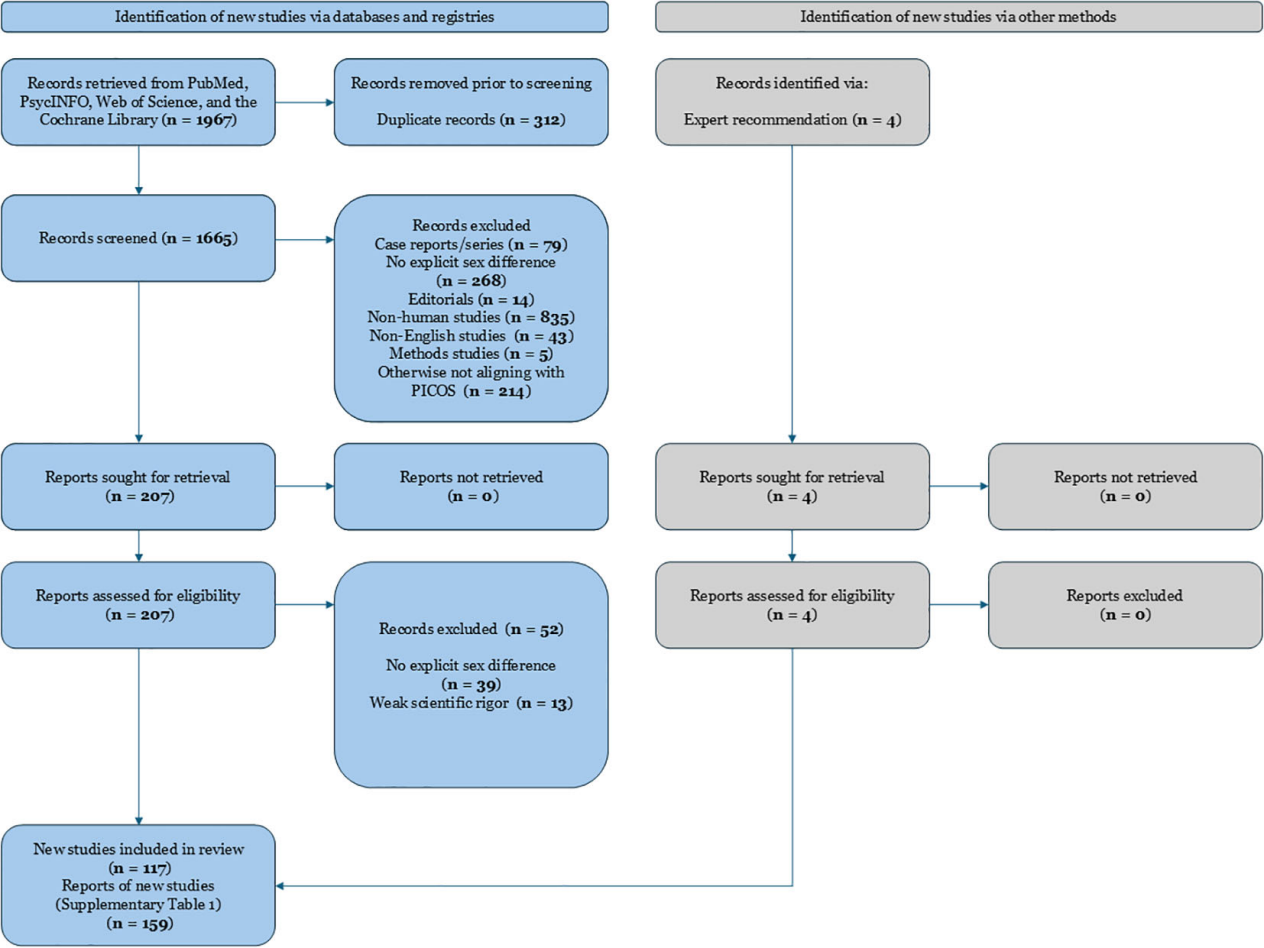
PRISMA 2020 flow diagram depicting the study selection process for a systematic review examining sex differences in schizophrenia. Records were identified from searches conducted in PubMed, PsycINFO, Web of Science, and the Cochrane Library. After removing duplicates and irrelevant records, full-text articles were assessed for eligibility. Main reasons for exclusion at the full-text stage included absence of explicit examination of sex differences, non-human studies, weak scientific rigor, non-English language, and studies not meeting PICOS criteria. Ultimately, 159 studies were included in the systematic review.

This review was conducted following a systematic approach to identify and synthesize studies on sex differences in schizophrenia. A comprehensive literature search was performed in major databases including PubMed, PsycINFO, Web of Science, and the Cochrane Library. The search strategy combined keywords related to schizophrenia with terms denoting sex or gender differences and specific outcome domains. For example, we used queries such as “schizophrenia AND sex differences AND treatment response,” “schizophrenia AND gender AND symptomatology,” and “schizophrenia AND sex AND adverse effects” (7, 22). Reference lists of relevant articles and reviews were also hand-searched to ensure inclusion of all pertinent studies. The search covered articles published up to January 2025, with an emphasis on contemporary research (the last 10 years) while including seminal older studies for context. Information on the exact search procedure can be found in **Figure 1**.

Inclusion criteria were defined according to the PICOS framework (Population, Intervention, Comparison, Outcome, Study design). We included peer-reviewed human studies (clinical trials, cohort studies, cross-sectional analyses, and meta-analyses) that explicitly examined differences between men and women with schizophrenia. Both chronic schizophrenia and first-episode psychosis populations were considered. Relevant outcomes included neurobiological measures (e.g., neuroimaging findings, genetic or hormonal factors), treatment outcomes (antipsychotic efficacy, response rates, relapse, and adherence), clinical symptomatology (positive, negative, and affective symptom profiles, age at onset, etc.), and adverse treatment effects (side-effect prevalence and severity). We excluded case reports, small case series, and studies conducted in animal models. Non-English articles, conference abstracts, and non-peer-reviewed sources were also excluded to ensure quality and reliability.

After removing duplicates, two reviewers (I.V. and V.K.) independently screened titles and abstracts for relevance. Studies that appeared to meet inclusion criteria were retrieved in full text and assessed for eligibility. Disagreements in study selection were resolved through discussion and consensus, consulting a third reviewer expert in the field if needed. Data extraction was performed using a standardized form, capturing key study characteristics and outcomes stratified by sex. We paid particular attention to extracting results on treatment response differences (e.g., differential efficacy or dosing requirements), symptom differences (such as variations in symptom severity or subtype by sex), and adverse effect profiles in men vs. women. The quality of included studies was appraised using criteria appropriate to each study’s design (for example, risk of bias tools for randomized trials and observational studies). We identified 159 relevant and recent studies that met inclusion criteria, encompassing a diverse range of methodologies and patient populations. Because this is a qualitative systematic review, no meta-analytic statistical pooling was performed; instead, findings are narratively synthesized. Our reporting follows PRISMA guidelines for systematic reviews, and a PRISMA flow diagram of study selection is provided (see **Figure 1**).

## Results

The findings are organized below into four main subsections: Neurobiological Differences, Treatment Response, Symptomatology, and Adverse Effects, reflecting the domains of interest.

### Neurobiological differences

#### Brain structure and neurodevelopment

Numerous studies have probed whether schizophrenia’s well-documented brain abnormalities manifest differently by sex. Overall, men and women with schizophrenia both exhibit structural brain changes relative to healthy controls, but there are subtle distinctions. For instance, evidence suggests that sex differences in brain morphology (such as volumetric differences in certain regions) and function seen in the general population tend to persist in patients with schizophrenia (6, 23). A recent large-scale neuroimaging meta-analysis found that man and woman schizophrenia patients showed divergent shape characteristics in deep brain structures that mirrored normal sexual dimorphism, implying that inherent neurodevelopmental sex differences are preserved to some degree in the illness (24). At the same time, some region-specific differences have been noted: the inferior parietal lobe volume reported that men with SZ lost the normal left-greater-than-right asymmetry seen in healthy men, whereas women did not exhibit this specific alteration (25). Women with SZ had decreased baseline activation of the dlPFC than men, a region that is involved in higher cognitive functions like working memory, flexibility, and planning (26). These findings provides further evidence that sex differences in brain morphology and function may contribute to the differing clinical expression of schizophrenia in men and women (27). It should be noted, however, that results across neuroimaging studies are not entirely consistent (25, 28). Differences that are observed often occur in regions that normally show sexual dimorphism (for example, limbic and cortical areas influenced by sex hormones), but normal sexual dimorphism in white matter is reverted in high risk individuals for schizophrenia emphasizing the neurodevelopmental aspect of this disorder (29).

#### Genetic and molecular factors

Emerging research indicates that genetic risk factors for schizophrenia might have sex-specific effects. A notable example concerns the complement component 4 (C4) gene, which is involved in synaptic pruning, which in turn is involved in the neurobiology of schizophrenia. Recent genetic analyses have suggested that certain C4 risk haplotypes for schizophrenia have a larger impact in men than in women, potentially contributing to the greater frequency of men in early-onset schizophrenia (30). Sex differences in immune function and complement protein levels (with adult women generally showing lower complement levels than men) might moderate the effect of these gene variants (31). Beyond C4, other gene-by-sex interactions have been explored. For instance, one study on the MTHFR gene (involved in folate metabolism) found that a functional polymorphism was associated with differences in symptom severity in men with SZ but not in women (12). Another investigation identified a sex-specific association involving the BDNF (brain-derived neurotrophic factor) gene: a particular BDNF variant predicted antipsychotic-induced weight gain in men but not in women, whereas low BDNF protein levels were linked to weight gain in women (32). These examples underscore that the genetic architecture of schizophrenia risk and treatment response may not be uniform across sexes. Epigenetic mechanisms and gene expression profiles also show sex-specific patterns in schizophrenia, although research in this area is still developing (33).

#### Hormonal differences

Perhaps the most evident biological disparities between men and women with schizophrenia involve gonadal hormones. The estrogen hypothesis of schizophrenia posits that estrogen confers a protective effect in women, delaying onset and attenuating symptoms until endogenous estrogen levels decline (34). Consistent with this, premenopausal women experience later onset and often less severe negative symptoms than age-matched men (35). Conversely, illness risk rises in women during low-estrogen phases (such as postpartum and postmenopause), aligning with the idea that estrogen withdrawal can precipitate or worsen psychosis (7, 36). In men, the role of testosterone and other androgens in schizophrenia is complex, with some evidence that low testosterone is linked to negative symptom severity, although findings are mixed (37). Recent clinical studies have directly measured sex hormone levels in first-episode patients to elucidate differences at illness onset. In one study of antipsychotic-naïve young patients from 2024, researchers found pronounced sex-specific hormonal disruptions at first episode (38). Young men with schizophrenia had significantly elevated estradiol levels compared to healthy men, while young women with SZ showed abnormal levels of multiple hormones relative to healthy women (38). These results indicate an overall gonadal hormone imbalance in early psychosis, with men uniquely exhibiting high estrogen and women exhibiting broader endocrine dysregulation. Hormonal fluctuations may also modulate symptoms during the course of the illness; for example, some women experience psychotic symptom exacerbation premenstrually when estrogen temporarily dips, and small trials of estrogen replacement or selective estrogen receptor modulators have shown a potential antipsychotic benefit in women (39).

**Figure 2:**
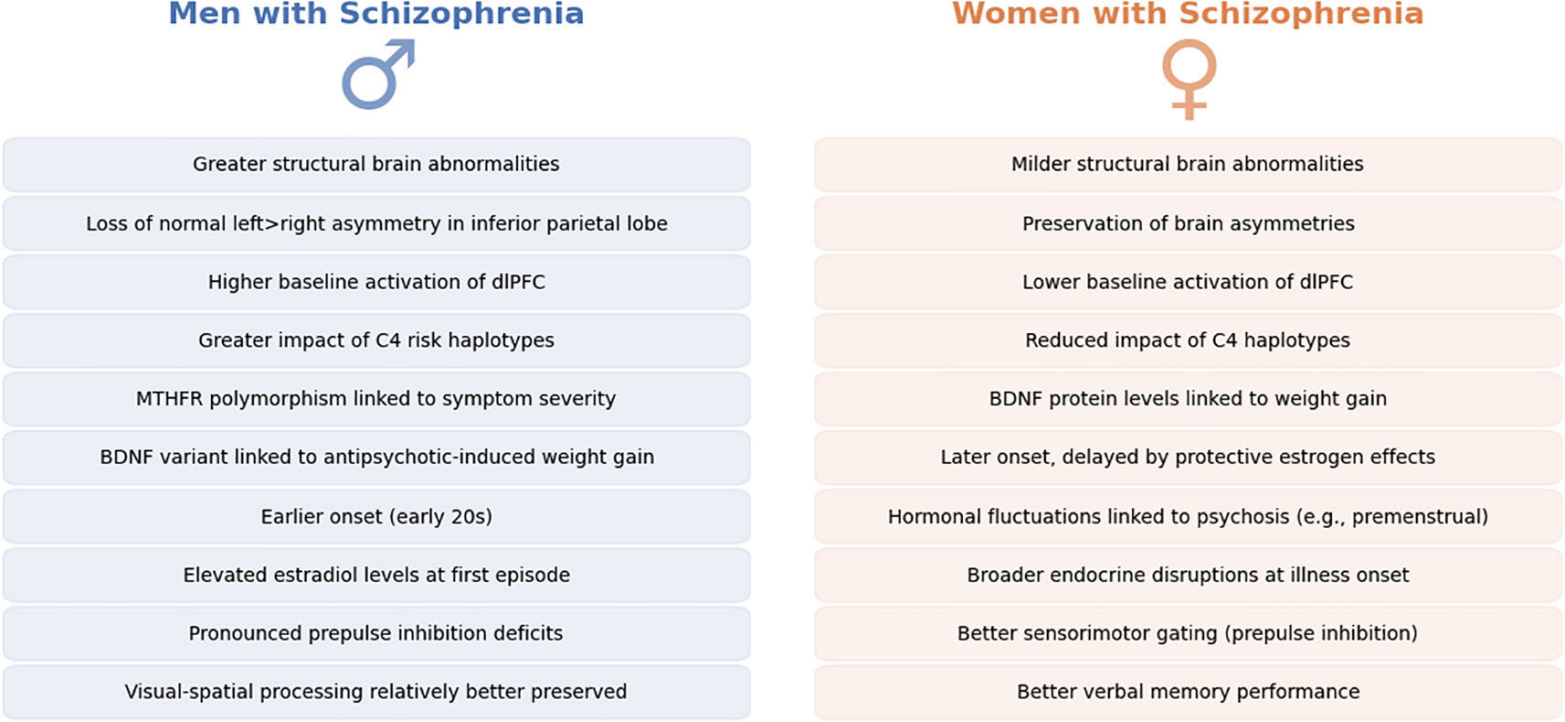
Neurobiological sex differences in Schizophrenia. This figure provides a side-by-side comparative overview of neurobiological differences observed between men (left, in blue) and women (right, in orange) diagnosed with schizophrenia. Each panel summarizes key findings from neuroimaging, genetic, hormonal, and cognitive studies reported in recent literature. Differences noted include variations in brain morphology, such as structural abnormalities and functional activation patterns, differential genetic influences, hormonal imbalances affecting disease onset and progression, and subtle distinctions in cognitive processing between sexes.

Likewise, cognitive function profiles show subtle differences: some data indicate women with schizophrenia perform better on verbal memory tasks whereas men may have an advantage in visual-spatial processing, mirroring normal sex dimorphisms, though both sexes are impaired relative to controls (40). Notably, cognitive impairment in schizophrenia is a strong predictor of long-term functional outcome, making these sex-specific differences particularly relevant for designing targeted rehabilitation programs (41). Overall, while the core neuropathology of schizophrenia is shared by men and women, measurable biological differences in brain structure, genetics, and hormones likely underlie the distinct clinical profiles observed across sexes (42). Main findings in neurobiology are summarized in **Figure 2**.

### Treatment response

#### Antipsychotic efficacy and dosing

A growing literature indicates that men and women can respond differently to antipsychotic medications. Notably, pharmacokinetic differences between sexes often result in higher antipsychotic blood levels in women for a given dose. Women tend to have lower activity of certain drug-metabolizing liver enzymes (such as CYP1A2) and a higher body fat proportion, which can lead to slower clearance of antipsychotics (43). One clinical trial explicitly examining sex differences in antipsychotic treatment (the Best Strategy in Treatment-Resistant Schizophrenia [BeSt InTro] study) found that women achieved significantly higher plasma concentrations of various antipsychotics than men when treated at the same dose. In that study, dose-corrected serum levels of amisulpride were approximately 72% higher in women than in men, and levels of aripiprazole were about 56% higher in women, indicating markedly slower drug metabolism in women. Consequently, men often require higher doses to obtain therapeutic effects; for example, men with SZ needed moderately higher doses of aripiprazole to achieve comparable symptom control (44). By contrast, women face a risk of relative “overdosing” if dosages are not adjusted, given their greater drug exposure for the same dose (45).

#### Therapeutic efficacy

In terms of therapeutic efficacy, some antipsychotic agents appear to work better in one sex versus the other. For instance, amisulpride (a dopamine D2/D3 blocker) was found to be highly effective in men, producing a faster reduction in psychotic symptoms in men with SZ, whereas women derived less benefit from this drug (44, 46), 2007). Hoekstra et al. showed that men with SZ randomized to amisulpride had a significantly more rapid and robust symptom improvement than women (p = 0.003 for sex difference in speed of response). Moreover, amisulpride outperformed other medications (olanzapine and aripiprazole) in men but not in women (44). This suggests a potential sex-by-medication interaction where amisulpride is a preferential option for men with SZ. However, another study failed to uncover any sex differences in amisulpride treatment efficacy between men and women with SZ (47). Evidence indicates that women may respond more favorably to certain antipsychotics, such as paliperidone, particularly in first-episode populations. Women first-episode of psychosis (FEP) patients treated with paliperidone have been noted to achieve higher remission rates and symptom reduction than their men counterparts in some trials (16). Another study focused on improvement of symptomatology and found olanzapine to be superior to other antipsychotics in women after FEP (48) possibly due to differentiated metabolization rates (14). Similarly, clozapine, the prototypical treatment for treatment resistant refractory schizophrenia, has been reported in some cohorts to yield slightly higher response rates in women—possibly related to women’s higher clozapine blood levels for a given dose and clozapine’s interaction with estrogen—though findings on clozapine efficacy by sex are mixed (49, 50).

#### Symptom trajectory and outcomes

Beyond acute efficacy, long-term outcomes of treatment also might be influenced by sex. Women generally have better short-term outcomes early in schizophrenia—for example, a higher likelihood of achieving remission of positive symptoms in the first year (51). One large observational study noted that at one-year follow-up, a greater proportion of women were in remission and had improved functioning compared to men with equivalent treatment (52). This early advantage may reflect both biological factors (e.g., estrogen’s effects) and psychosocial factors (e.g., better premorbid skills aiding recovery). Adherence during the maintenance phase can also differ: some reports suggest men with SZ are slightly more likely to discontinue medication or relapse early, perhaps due to higher rates of substance abuse and poorer insight, whereas women, when not hampered by side effects, may adhere more consistently (53). Indeed, one analysis found that men were more prone to relapse after a medication dose reduction or discontinuation, whereas women maintained relapse-free stability longer when reducing antipsychotic dosage (54). This could be related to women’s generally lower dosing requirements; reducing the dose might affect men more adversely if they were just at the threshold of efficacy. On the other hand, among homeless patients with schizophrenia, one study found that homeless women had poorer treatment adherence than homeless men, possibly due to differing social support or victimization factors (55).

When examining long-term outcomes, some longitudinal studies have found that over decades of illness, outcome differences narrow or even reverse. For instance, a 10-year follow-up study reported that while women had better functional outcomes in early years, by the decade mark, men and women showed similar levels of clinical improvement and functional status on maintenance treatment. In the same study, there is also evidence that after mid-life (post-menopause), women’s illness severity can worsen relative to men’s, eroding earlier gains (56). A Finnish cohort study observed that women’s hospitalization rates and symptom severity increased after age 45, whereas men (who had already experienced a chronic course) changed little, resulting in mostly equivalent outcomes in later life (57). Such findings highlight that sex differences in schizophrenia are most pronounced in the early and mid-phases of illness, while late-life schizophrenia may converge across sexes. Nevertheless, some differences persist: for example, insight into illness and willingness to seek treatment have generally been found to be better in women with SZ, which can positively influence long-term adherence and outcomes (58). Women with SZ often engage more in psychosocial treatments and have stronger family support, factors linked to better prognosis. In contrast, men have higher rates of behaviors that complicate treatment (e.g., aggression, substance abuse, homelessness), which can lead to more frequent relapses or rehospitalizations (41).

#### Clinical management implications

**Figure 3:**
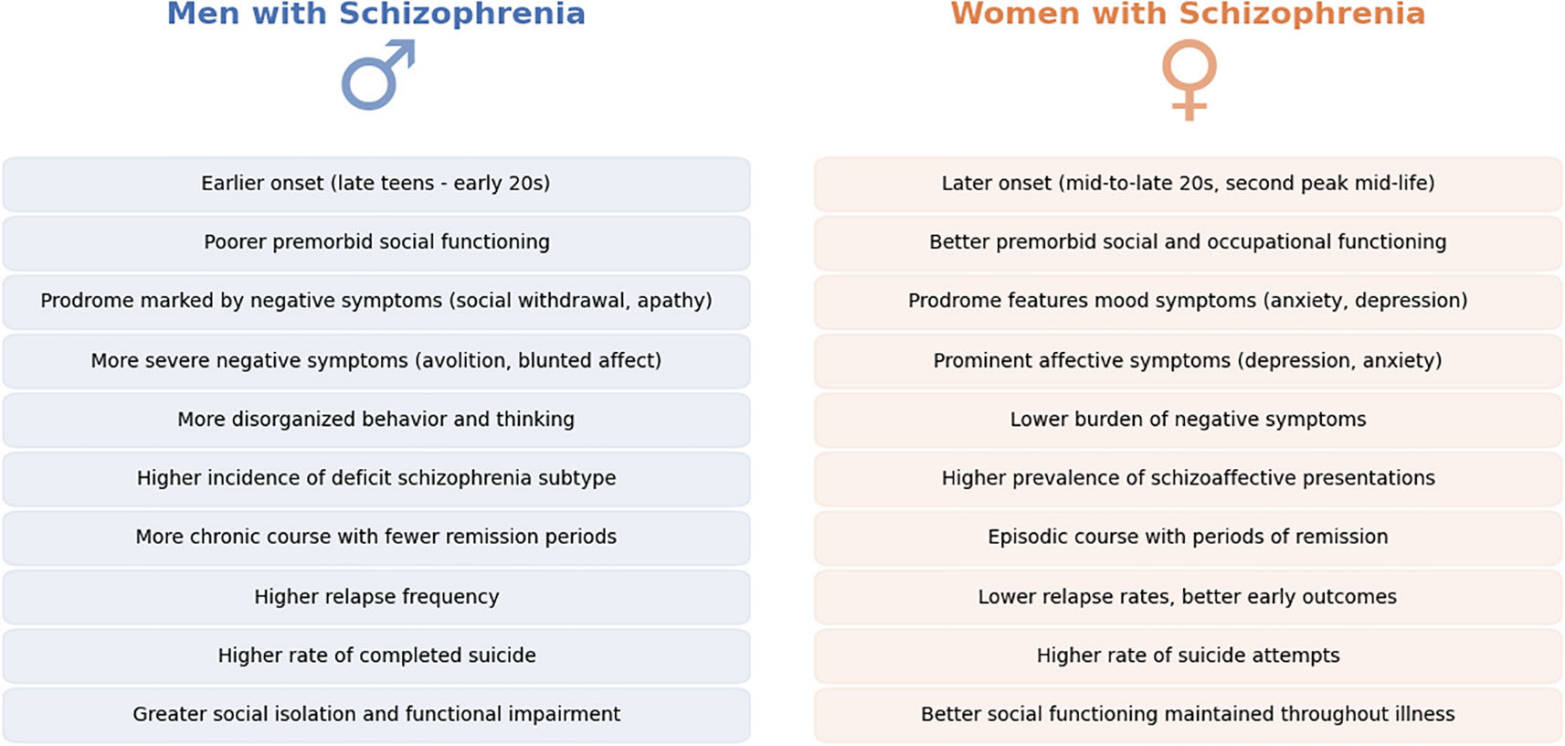
Sex differences in Schizophrenia symptomatology and illness course. This figure presents a comparative summary of key symptomatic and clinical course differences between men (left, in blue) and women (right, in orange) diagnosed with schizophrenia. Observations drawn from the literature highlight disparities in the age of illness onset, premorbid functioning, prodromal symptom profiles, predominant symptom types (e.g., affective versus negative symptoms), and patterns of illness progression and remission. The noted differences underscore variations in clinical expression and illness trajectory between sexes. Future clinical practices may benefit from systematically incorporating these symptom and illness-course sex differences, facilitating earlier and more personalized interventions tailored to each patient's specific needs.

The differential response patterns suggest that clinicians should consider sex when choosing and dosing antipsychotics. Women may achieve the same therapeutic benefit at lower doses and are at risk of overtreatment if dosages are not adjusted (due to higher blood levels), as previously mentioned (44). Conversely, men might require more aggressive dosing or preferential use of high-potency antipsychotics to reach symptom control (49, 59). Some experts have called for sex-specific treatment guidelines, noting that current prescribing practices often neglect these differences (53). For example, considering a trial of adjunctive estrogen or selective estrogen receptor modulators in women with persistent negative symptoms is an area of active investigation, given some positive trials showing estrogen’s augmentation effect (39). In men, ensuring adequate dosing and addressing comorbid substance use (which is more prevalent in men) are key for optimizing outcomes (6, 8, 60). Importantly, the goal is not to treat men and women wholly differently but to personalize treatment in recognition of sex-related tendencies. Both pharmacotherapy and psychosocial interventions (e.g., targeted compliance programs or gender-specific support groups) may benefit from tailoring to the unique needs and strengths of each sex. In summary, while antipsychotics remain the cornerstone of schizophrenia treatment for both men and women, accumulating evidence underscores that a “one-size-fits-all” approach may be suboptimal. Appreciating sex differences in pharmacokinetics and treatment response can guide more nuanced, effective management strategies for schizophrenia patients of each sex (61, 62). Treatment response sex-differences are presented in **Figure 3**.

### Symptomatology

#### Age at onset

One of the most robust sex differences in schizophrenia is the age at illness onset. Men develop schizophrenia on average 3–5 years earlier than women (1, 63). Epidemiological studies consistently show a peak onset for men in late adolescence to early twenties, whereas the peak for women is in the mid-to-late twenties with a second smaller peak around mid-life (35). In a large cohort study the mean age of onset was approximately 23.7 years in men versus 27.6 years in women, a statistically significant gap (64). This delay in women has been attributed largely to the protective effect of estrogen during childbearing years (7). Interestingly, after women reach menopause (when estrogen levels drop), their incidence of schizophrenia can rise and sometimes even approach that of men, contributing to a later second peak of onset in women. Men, having no equivalent hormonal protection, experience their highest risk in early adulthood. The later onset in women often allows for better development of social and occupational skills before illness strikes, partially explaining their superior premorbid functioning (65).

#### Premorbid and prodromal features

The period before the onset of frank psychosis also shows sex-specific patterns. Men with SZ often exhibit more social withdrawal, academic difficulties, and schizoid or schizotypal traits in adolescence prior to diagnosis (64). They are more likely to have had behavioral problems or subtle developmental delays noted in childhood. Women, on the other hand, tend to have comparatively normal social development premorbidly; many have had stable relationships or employment prior to illness, reflecting better psychosocial adjustment. In clinical terms, men have a lower level of social development at illness onset, which subsequently impedes their further social maturation (66). This can lead to the often-observed outcome that schizophrenia’s social consequences (unemployment, isolation) are more severe in men. During the prodromal phase (the months or years of subtle symptoms before psychosis), some studies suggest men exhibit more negative symptoms (apathy, blunted affect) and unusual behavior, whereas women prodromally may experience more mood symptoms (e.g., anxiety, mild depression) or nonspecific physical complaints (67, 68). One interesting finding is that women in high-risk prodromal states are more likely to receive early interventions (such as antipsychotic treatment) than men (69). This could be because women’s prodromal symptoms bring them into contact with health services sooner, or clinicians might be more proactive in treating young women at risk. In any case, early intervention studies have noted that despite similar criteria, fewer men engage in preventive treatment during the prodrome, possibly contributing to a more abrupt and severe onset in men.

#### Clinical presentation and symptom profile

Once the illness is established, men and women can show distinct symptom profiles. Positive symptoms (hallucinations, delusions) are a prominent feature in both sexes, but some research suggests that women may have more affect-laden or auditory hallucinations, whereas men more often exhibit paranoid delusions (70, 71). In contrast, men with SZ exhibit more severe negative symptoms on average – such as flattened emotional expression, avolition, and social withdrawal and have different risk factors for developing this symptomatology than women (72–74). Men also more frequently meet criteria for the deficit subtype of schizophrenia (characterized by primary, enduring negative symptoms). For example, studies have found that deficit schizophrenia is disproportionately diagnosed in men, whereas women are more often diagnosed with non-deficit schizophrenia with more mood reactivity (75). Depressive symptoms and anxiety are reported more often by women with schizophrenia (3, 5). Women’s psychotic episodes commonly include prominent affective components (schizoaffective presentations are more frequent in women). Suicidality is another aspect with sex differences: while the overall suicide attempt rate in schizophrenia is high for both sexes, women may attempt suicide at slightly higher rates—often related to depressive symptoms—whereas men have a higher rate of completed suicide, potentially linked to greater social isolation and substance misuse (6, 63).

Some symptom differences emerge in specific contexts. For instance, the diagnostic subtype of schizophrenia has historically shown gender trends: earlier studies indicated that paranoid schizophrenia might be relatively more common in women (who often have later onset, more organized delusions), whereas disorganized or deficit presentations were more common in men (62) this has not been a universal finding; a study of first-episode patients in China found no sex difference in subtype at initial presentation, but in chronic samples, women were slightly more represented in paranoid subtype while men in negative/disorganized subtypes (41). Additionally, catatonic symptoms, though rare, have been reported more in men in some series. On the other hand, cognitive symptoms (neurocognitive deficits in memory, executive function) are a core feature for most patients and generally do not show large sex differences in magnitude – both men and women with schizophrenia suffer cognitive impairment relative to healthy controls. If anything, women’s slightly better premorbid verbal skills may translate to marginally better performance on verbal memory tests than men with schizophrenia, but when matched for education, cognitive profiles are largely comparable across sex (1, 64, 76).

#### Course of illness and recovery

**Figure 4:**
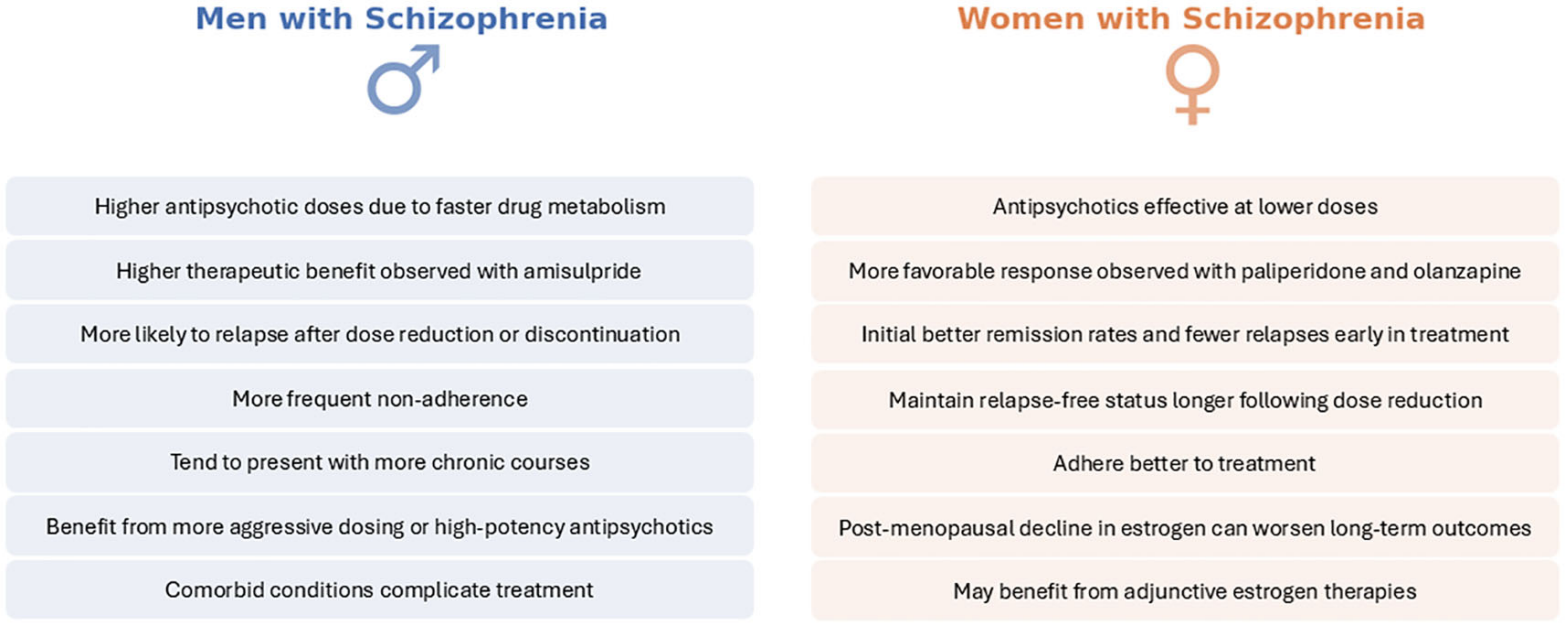
Sex differences in treatment response. This figure provides a concise side-by-side comparison of treatment response betwet men (left, blues) and women (right, orange). Key findings hghlight variations in antipsychotic efficiency and dosing, therapeutic efficiency and symptom trajectory and outcomes. Considering these sex-specific patterns during clinical assessment and therapeutic planning may enhance precision and effectiveness in managing schizophrenia across diverse patient populations.

Women not only present later but often have an episodic course with periods of remission, whereas men more often have a continuous course with residual symptoms. Clinical studies have observed that women are more likely to achieve remission of positive symptoms on treatment, especially in early and mid-life (51, 77). One large observational study noted that at one-year follow-up, a greater proportion of women were in remission and had improved functioning compared to men with equivalent treatment (52). This early advantage may reflect both biological factors (e.g., estrogen’s effects) and psychosocial factors (e.g., better premorbid skills aiding recovery). Adherence during the maintenance phase can also differ: some reports suggest that while men adhere better they are more likely to relapse early, perhaps due to higher rates of substance abuse and poorer insight, whereas women, possibly hampered by side effects, adhere less consistently (78, 79). On the same note, specific subpopulations continue this pattern – for example, among homeless patients with schizophrenia, one study found that homeless women had poorer treatment adherence than homeless men (55). **Figure 4** highlights key differences in symptomatology between men and women.

### Adverse effects

#### Metabolic side effects

One of the most concerning and well-studied long-term side effects of antipsychotic therapy is metabolic syndrome (weight gain, dyslipidemia, glucose intolerance). Studies have shown notable sex differences in metabolic outcomes. Women appear more significantly more vulnerable to antipsychotic-induced weight gain on certain medications (44, 80, 81). For example, in the previously mentioned randomized trial, women receiving amisulpride had significantly greater increases in body mass index (BMI) compared to men on the same treatment (44). Women’s BMI rose more and faster, especially with prolactin-elevating antipsychotics, suggesting a higher propensity for weight gain. Other research has found that atypical antipsychotics like clozapine and olanzapine confer a remarkably higher risk for metabolic dysfunction in women than in men. Women with SZ on these medications had higher odds of developing significant weight gain, hyperglycemia, and dyslipidemia relative to men with SZ on the same drugs (40). One proposed reason is that estrogen and other hormones in women might promote adipose deposition, or that women’s lower lean body mass results in greater effective drug concentration, amplifying metabolic side effects.

On the other hand, some data suggest that men are not immune to metabolic issues and may exceed women in certain parameters. For instance, a naturalistic study found that men with SZ were more likely to show elevations in cholesterol (especially LDL) and fasting glucose under long-term antipsychotic treatment, whereas women did not exhibit as steep an increase in those measures (82). Additionally, because men are more prone to abdominal (visceral) obesity when they gain weight, men with SZ might have a higher risk of cardiometabolic complications even if the degree of weight gain is similar. Indeed, one analysis reported that men with schizophrenia had a higher incidence of diabetes and dyslipidemia as well as more visceral fat tissue than women over a follow-up period, despite women gaining more weight in some cases (83). This highlights that the relationship between sex and metabolic side effects is complex: women gain more weight overall, but men might experience more adverse metabolic consequences per unit weight gained. It is worth noting that at least one study found no significant sex difference in the prevalence of metabolic syndrome among chronic patients – both sexes had high rates, likely due to universally poor lifestyle and antipsychotic effects (84). Thus, while trends exist with women often highlighted as higher risk for weight gain, clinicians should monitor metabolic health closely in all patients. Still, special vigilance is warranted for young women starting second-generation antipsychotics, as they can rapidly gain weight and develop metabolic syndrome unless preventive measures (diet, exercise, or preventative medication) are implemented (40).

#### Endocrine side effects (prolactin and sexual)

Prolactin elevation is a common antipsychotic side effect, especially with drugs like typical antipsychotics or risperidone and amisulpride that strongly block dopamine D_2_ receptors in the pituitary. Women are generally more susceptible to hyperprolactinemia on these medications (85). The high affinity for the dopamine receptor and subsequent agonism promotes prolactin production and the lack of 5-HTergic action does not inhibit it. Evidently, women more frequently experience hyperprolactinemia-related side effects such as menstrual irregularities (amenorrhea or oligomenorrhea), galactorrhea (milk discharge), and fertility issues (86). Many women with SZ on risperidone or amisulpride report loss of menstrual periods due to elevated prolactin. In contrast, men can also develop hyperprolactinemia, but the clinical sequelae differ – high prolactin in men may cause sexual dysfunction (e.g., decreased libido, erectile problems) and sometimes gynecomastia, though gynecomastia can occur in women as well (87, 88).

Despite such mixed findings, certain specific sexual side effects do show sex specificity. Erectile dysfunction and ejaculation difficulties are side effects unique to men, and antipsychotics—especially those raising prolactin or with alpha-adrenergic blocking properties—can cause impotence in a substantial fraction of men with SZ. Women do not experience erectile problems, but they may experience decreased sexual desire and arousal, as well as anorgasmia, which can be equivalent in impact (17, 19). Some evidence suggests that women are more likely to notice and be distressed by sexual side effects, potentially because men in this population may already have lower sexual activity due to negative symptoms or social factors. Also, hyperprolactinemia’s consequences like osteoporosis can differ: prolonged prolactin elevation leads to hypogonadism in both sexes, contributing to reduced bone density (89). Interestingly, one study found that men with SZ on prolactin-raising antipsychotics had higher rates of osteoporosis and low bone mineral density than women, which might reflect that clinicians are less likely to intervene for hypogonadism in men (90). This indicates that both sexes have unique vulnerabilities: women to reproductive side effects, and men to quietly developing bone loss or sexual dysfunction that they may not report.

Importantly, newer antipsychotics with partial dopamine agonism (such as aripiprazole) or those that spare prolactin (like quetiapine) can mitigate these endocrine side effects (91). Studies have shown that switching a patient (especially a woman with amenorrhea) from risperidone to a prolactin-sparing agent often restores normal menstrual function (86). Aripiprazole, in particular, can reduce prolactin levels in both men and women when added to or replacing a prolactin-elevating antipsychotic, with equivalent efficacy in normalizing prolactin across sexes (91). Thus, managing prolactin side effects may involve sex-specific considerations (e.g., ensuring premenopausal women maintain regular menses and bone health, and monitoring testosterone levels in men with long-term hyperprolactinemia).

#### Cardiovascular side effects

Antipsychotics can prolong the cardiac QT interval, raising the risk for a polymorphic ventricular tachycardia, torsade de pointes, an arrythmia which can be lethal if unmanaged. Female sex is a known risk factor for drug-induced QT prolongation in the general population (92, 93). This extends to the adverse effect profile for medications that alter heart repolarization such as antipsychotics. Women inherently have slightly longer baseline QT intervals than men, and estrogen may further lengthen cardiac repolarization (94). Research in schizophrenia patients indicates that severe QT prolongation (QTc > 500 ms) is observed more frequently in women on antipsychotics than in men (95). For example, a study in patients on long term antipsychotic therapy found that the prevalence of significant QTc prolongation was higher in women, despite similar drug regimens (96). Ziprasidone and clozapine have the highest rates of QT prolongation and cardiac toxicity (97) and ziprasidone was more frequently discontinued in women than in men with SZ (98), while women with SZ under clozapine treatment exhibit a more pronounced QT prolongation effect than men (99). This sex difference in cardiotoxicity risk is important for medication selection and ECG monitoring. Clinical guidelines usually recommend more cautious dose titration and periodic ECGs in women, especially if they are on multiple QT-prolonging drugs or have other risk factors (100). Men are not exempt from antipsychotic cardiac effects, but statistically, torsade de pointes has been reported more in women (101).

#### Neurological side effects

Antipsychotic-induced extrapyramidal symptoms (EPS), such as parkinsonism, tremor, and tardive dyskinesia (TD), also show some sex differences. Acute EPS (such as drug-induced parkinsonism) may be slightly more frequent in men, though evidence is not uniform (102, 103). Historically, TD was found to be more common in older women with schizophrenia, especially those who had long exposure to first-generation antipsychotics (104). Postmenopausal women were at highest risk for TD, possibly because estrogen has a dopamine-blocking effect; when estrogen levels drop, dopamine receptor supersensitivity (a cause of TD) might be unmasked (105). In the era of second-generation antipsychotics, TD incidence has declined, but some recent studies still find woman sex as a risk factor for tardive syndromes, particularly in those over 45 (57). On the other hand, acute EPS (such as drug-induced parkinsonism) may be slightly more frequent in men, though evidence is not uniform (103). One reason could be that men often receive higher doses and more high-potency antipsychotics (e.g., haloperidol) which cause EPS, whereas women more often receive atypical antipsychotics at moderate doses. Additionally, akathisia (restless agitation) has been reported more in men with SZ in some trials, potentially related to higher baseline motor activity or co-occurring substance use (102). These observations imply that long-term treatment plans must consider the differing risks: clinicians should monitor movement disorders closely in women and consider dose adjustments or switching medications if TD emerges, whereas men might benefit from adjunctive therapies aimed at mitigating acute EPS.

#### Other adverse effects

Some side effects are idiosyncratic but have noted sex biases. For example, it has been shown that women tend to exhibit less adverse effects from clozapine (49, 106). Blood dyscrasias like eosinophilia or agranulocytosis from clozapine have been reported more in one sex – men on clozapine had higher rates of neutropenia or other blood dyscrasias than women. The same large cohort study also reported that men had an elevated risk of cardiomyopathy and myocarditis (107). Liver enzyme elevations on certain antipsychotics might occur more in men (possibly due to higher alcohol use) (108). Sedation is a side effect that both men and women report, but some surveys suggest women may be more sensitive to its impact on daily functioning and quality of life (109). In contrast, men, who often are on higher doses, might experience more pronounced sedation when the dose is increased. Adherence-related effects (such as subjective feelings of dysphoria or cognitive dulling) could impact sexes differently: some evidence suggests that women are more likely to discontinue medication due to weight gain and menstrual side effects, whereas men are more likely to stop due to extrapyramidal side effects, sexual side effects or a subjective sense of emotional blunting (78, 88).

**Figure 5:**
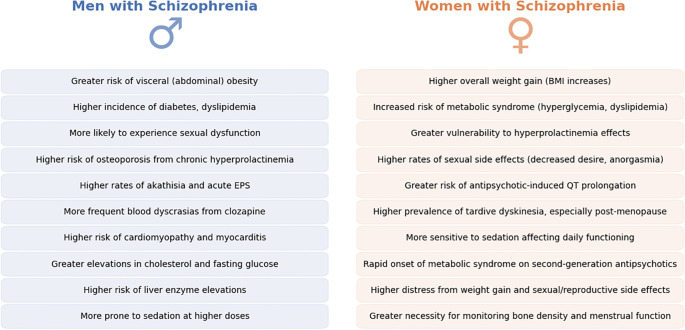
Sex differences in antipsychotic-induced adverse effects in Schizophrenia. This figure summarizes key sex-specific adverse effects associated with antipsychotic treatment in schizophrenia, contrasting men (left, blue) and women (right, orange). Differences span metabolic, endocrine, cardiovascular, and neurological domains, illustrating that men and women experience unique vulnerabilities when exposed to antipsychotic medications. Recognizing and anticipating these sex-related differences in clinical practice could facilitate more effective patient counseling, targeted monitoring, and proactive management of treatment-related complications, ultimately improving patient adherence and quality of life.

In summary, the adverse effect profiles of antipsychotic treatment in schizophrenia demonstrate clear sex differences. Women are more prone to weight gain, metabolic syndrome, hyperprolactinemia, sexual/reproductive disturbances, and QT prolongation whereas men are more prone to certain metabolic abnormalities (like dyslipidemia) and may experience more pronounced extrapyramidal symptoms. These differences necessitate sex-specific monitoring and management. For women, it is crucial to monitor weight, blood glucose/lipids, menstrual function, and bone density, and to consider adding a partial dopamine agonist or switching to prolactin-sparing and weight-neutral antipsychotics if problems arise (91, 110). For men, aggressive management of cardiovascular risk factors (given higher smoking rates and cholesterol findings) and vigilance for EPS and sexual side effects are key (111). With newer treatment options (e.g., partial agonists, statins, adjunct metformin for weight control), clinicians have tools to mitigate many side effects. The overarching principle is that being aware of a patient’s sex can guide anticipation of likely side-effect challenges—for instance, a young woman might need education about potential weight gain and contraceptive considerations, while a young man might require counselling on avoiding substances that exacerbate metabolic side effects. Tailoring side-effect management to sex not only improves quality of life but also enhances treatment adherence (112). Main findings are summarized in **Figure 5**.

## Discussion

This systematic review highlights significant sex differences in the neurobiology, clinical presentation, treatment response, and side-effect burden of schizophrenia. The compiled evidence confirms that sex is a crucial modulator of schizophrenia, influencing everything from age of onset to medication efficacy. Evaluating and adjusting for these differences has concrete implications for optimizing patient care.

One of the clearest patterns emerging from the literature is that women have advantages in some aspects of schizophrenia outcome (later onset, higher early remission, better social functioning), whereas men have advantages in fewer domains but may respond poorer to certain treatments (50, 68). For example, consistent evidence indicates that women’s later onset and preserved social skills often translate into a less disruptive initial course and a higher likelihood of early remission (51). This suggests that if interventions are provided promptly, women have a strong capacity for recovery. In contrast, men’s earlier onset and greater negative symptom burden contribute to a more chronic course and lower likelihood of full remission (4, 6, 63). However, on the treatment side, some evidence suggests that men may derive disproportionate benefit from particular antipsychotics such as amisulpride (44) and generally tolerate higher doses (owing to faster metabolism) (45). This information could be leveraged in clinical practice: for men with severe psychosis, choosing a high-efficacy antipsychotic and titrating to the upper therapeutic range might be justified, whereas for a woman patient, an agent with a favorable metabolic profile and a lower starting dose may be preferable.

The role of sex hormones, especially estrogen, emerges as a unifying theme influencing many differences. Estrogen’s protective effect in women provides a compelling explanation for later onset, fewer negative symptoms, and a different neurobiological trajectory (with less early neurodevelopmental disruption) (7, 105). Adding estrogen or selective estrogen modulators (such as raloxifene) to existing medication has shown promise in ameliorating symptoms in women, particularly for refractory negative symptoms (39, 113). This suggests that hormonal modulation could become a viable sex-specific treatment strategy. It also underscores the need for clinicians managing women with schizophrenia to be mindful of hormonal phases; for instance, the peripartum period and menopause require proactive planning, as relapse risk may be heightened when estrogen levels decline (35). Conversely, the findings regarding men prompt questions about whether androgens or other man-specific factors could be harnessed or managed; for example, testing for decreased testosterone and correcting it might improve motivation and general symptomatology in men with SZ. Emerging evidence suggests that testosterone may have therapeutic relevance in men with schizophrenia, particularly for mitigating negative symptoms (114). Several studies have documented low or dysregulated testosterone levels in male patients, often associated with greater severity of avolition, anhedonia, and cognitive deficits. Small clinical trials have explored testosterone augmentation or supplementation with androgen precursors like DHEA, reporting modest but promising improvements in negative symptoms and mood (37). The proposed mechanisms include modulation of dopaminergic and glutamatergic signaling, neuroprotection, and enhancement of neuroplasticity. While these interventions have generally been well tolerated in the short term, more robust, long-term studies are needed to establish efficacy, identify responsive subgroups, and ensure safety.

Another key discussion point is how sex differences intersect with current treatment guidelines and whether guidelines should be revised. Currently, standard schizophrenia treatment does not differentiate by sex—dosing recommendations and medication choices are largely uniform. However, it has been suggested that a one-size-fits-all approach may be suboptimal. For example, given women’s higher antipsychotic blood levels and greater susceptibility to side effects, guidelines could recommend initiating treatment at lower doses for women with slower titration, while men might require standard or even higher doses to achieve comparable efficacy. In practice, implementing sex-specific considerations could involve multidisciplinary collaboration, such as involving endocrinologists or OB/GYN’s for women with SZ and addiction specialists for men with SZ, to tailor treatment to the unique needs of each sex.

Gaps in current research became apparent during our review. Despite a growing number of studies, several areas remain underexplored or yield conflicting data. For instance, neuroimaging findings on sex differences are inconsistent—some studies show significant differences in brain connectivity and morphology, while others do not (23, 27, 115). Similarly, the influence of sex chromosomes and epigenetic regulation on schizophrenia risk is still poorly understood. Although there is considerable knowledge about young and middle-aged patients, research on aging women with schizophrenia is scarce; as more women with schizophrenia live into older age, understanding how post-menopausal hormonal changes affect cognitive and functional outcomes becomes increasingly important (15). Moreover, while some evidence suggests that men are more likely to relapse after a medication dose reduction, the factors driving this difference remain ambiguous (54, 116). Such discrepancies highlight that sex interacts with many other variables—such as age, genetics, and environmental factors—in complex ways.

It should also be noted that most studies in our review were conducted in high-income settings, and low- and middle-income countries were underrepresented. This geographic bias is an important limitation, as sociocultural and healthcare disparities in LMICs may shape sex differences in schizophrenia outcomes (117). Resource-limited healthcare systems often lack access to newer adjunctive treatments (e.g., estrogen or hormonal therapies), potentially limiting benefits that disproportionately aid female patients. Moreover, traditional gender roles and differing stigma in many LMIC communities can alter disease trajectories – women with schizophrenia might be more likely to be hidden or receive family care at home, whereas men unable to fulfill expected roles may experience greater social marginalization (118). Such factors could modulate observed sex-specific outcomes, underscoring that our findings may not fully generalize to LMIC settings. The is a great and urgent need for more inclusive research and caution in extrapolating results to regions where women and men with schizophrenia face distinct sociocultural challenges.

This review focused explicitly on sex differences between men and women with schizophrenia; however, a significant limitation is the exclusion of non-binary and gender-diverse populations. Most schizophrenia research, including the studies reviewed here, predominantly adheres to a binary understanding of sex and gender. Recent literature emphasizes that such a binary framework contributes to an evidence gap regarding the prevalence, symptomatology, treatment response, and side-effect profiles in transgender, non-binary, and gender-diverse individuals with schizophrenia. Indeed, existing epidemiological data suggest an elevated risk of schizophrenia-spectrum disorders among transgender individuals, but findings remain inconsistent due to methodological variations and inadequate differentiation of transgender from non-binary or other gender-diverse identities (119). Moreover, Nolan et al. (120) note that this exclusion limits the generalizability of research findings, potentially leaving clinical guidelines inadequately tailored to gender-diverse individuals, who might possess unique clinical characteristics and treatment needs (120).

Sex differences in schizophrenia are shaped not only by biological factors but also by sociocultural and evolutionary influences. Gender roles, such as caregiving responsibilities and societal expectations, affect symptom expression, treatment adherence, and clinical outcomes (121). Women, often more socially connected due to traditional caregiving roles, tend to seek help earlier, leading to better early outcomes but may face added stress from these roles, which can impact relapse risk. In contrast, men, who experience more externalizing behaviors and substance use due to societal pressures to maintain self-reliance, often face worse outcomes. Evolutionary pressures further contribute to these differences: women’s stress response, influenced by estrogen and oxytocin, provides protection against psychosis until menopause, explaining their later onset and less severe early course. Men, with no such hormonal safeguard, show earlier onset and more chronic symptoms, reflecting evolutionary trade-offs linked to stress resilience and reproductive priorities (122). These sociocultural and evolutionary perspectives highlight the need for gender-specific interventions to optimize outcomes in schizophrenia.

In terms of clinical implications, it is more than evident that a more personalized approach to treating schizophrenia in men and women will be more efficient (123). For women with SZ, clinicians might emphasize weight management from the outset, choose antipsychotics with lower prolactin elevation if fertility is a concern, and monitor for mood fluctuations that may necessitate adjunctive treatments. For men with SZ, screening for substance use and ensuring adequate dosing (perhaps with depot formulations) could be prioritized. Additionally, both sexes might benefit from psychosocial interventions tailored to their unique challenges—such as social skills training for men and trauma-informed care for women. Ultimately, these differences underscore the necessity for future clinical trials to stratify outcomes by sex and for clinical guidelines to incorporate sex-specific recommendations.

## Conclusion

Men and women with schizophrenia differ significantly in epidemiology, clinical presentation, treatment response, and adverse effect profiles. Women tend to experience a later onset with better premorbid functioning and a more favorable early response to treatment, though they are at higher risk for metabolic and endocrine side effects. Men, by contrast, have an earlier onset, more pronounced negative symptoms, and often require higher doses for symptom control, but may be more vulnerable to substance use and certain neurological adverse effects. Recognizing these differences is crucial for tailoring treatment strategies and monitoring side effects in a sex-specific manner. Incorporating a sex-informed perspective into both clinical practice and future research will enhance personalized treatment approaches and ultimately improve outcomes for all individuals affected by schizophrenia.

### Future directions

Further research is needed to elucidate the underlying mechanisms of sex differences in schizophrenia, including basic neuroscience, large-scale genomics, neuroimaging, and hormonal studies. Longitudinal investigations tracking patients from the prodromal phase through chronic stages will be essential to understand how early advantages or disadvantages evolve over time. Additionally, intervention trials should be designed to detect sex-specific effects, thereby informing updated, gender-sensitive clinical guidelines. Tailoring both pharmacological and psychosocial treatments to account for sex differences represents a promising step toward truly personalized care in schizophrenia.

## Data Availability

The original contributions presented in the study are included in the article/**Supplementary Material**, further inquiries can be directed to the corresponding author/s.
