# Supplementary material for: Sex differences in schizophrenia: symptomatology, treatment efficacy and adverse effects
Source: Front Psychiatry. 2025 Jun 16;16:1594334. doi: 10.3389/fpsyt.2025.1594334 (PMC12206716; doi:10.3389/fpsyt.2025.1594334)
Supplement: Supplementary file 1 [file Table1.docx]

| Name | Year | Type | Sex-difference Compared | Sex-difference | Drug | Control Group | Main Finding |
| --- | --- | --- | --- | --- | --- | --- | --- |
| Abel, K. M., et al. | 2010 | Review | Symptomatology | Yes | No | No | Women with SZ often have a later age of onset, better premorbid functioning, and different symptom profiles. |
| Alberich, S., et al. | 2019 | Review | Treatment efficacy | Yes | No | No | Women with SZ might have a better response to clozapine and less adverse effects than men with SZ with equivalent plasma levels. |
| An, H., et al. | 2021 | Observational Study | Treatment efficacy | Yes | Yes | No | Women have lower blood concentration of olanzapine compared to men taking equivalent dosage. |
| Arranz, B., et al. | 2015 | RCT | Symptomatology | Yes | Yes | No | Substance consumption does not affect age of FEP in women. |
| Asif, U., et al. | 2018 | Observational Study | Adherence and response | Yes | Yes | No | Less side effects in men, but better adherence. |
| Bigos, K. L., et al. | 2008 | Clinical Trial | Metabolism of antipsychotics | Yes | No | No | Female sex leads to slower olanzapine metabolism. |
| Bo, Q., et al. | 2021 | RCT | Menstrual dysfunction and antipsychotics | Yes | Yes | No | Cognitive differences linked to schizophrenia symptom severity. |
| Bobes, J., et al. | 2007 | RCT | Adverse effects | No | Yes | No | No difference in metabolic syndrome prevalence. |
| Bobes, J., et al. | 2010 | RCT | Adverse effects | Yes | No | No | Women showed higher risk of adverse antipsychotic effects. |
| Bogenschutz, M. P. and H. George Nurnberg | 2004 | Clinical Trial | Treatment efficacy | Yes | No | Yes | Women exhibit a higher efficacy of olanzapine in a broad spectrum of disorders. |
| Boter, H., et al. | 2010 | RCT | Comorbidity with suicidality and substance use | Yes | No | No | Women were less likely to present comorbidity with substance use or suicidality. |
| Brand, B. A., et al. | 2023 | RCT | Estrogen in treatment | No | Yes | No | Some efficacy of estrogen activation in negative symptomatology. |
| Camsari, U., et al. | 2014 | Comparative Study | Diagnosis and treatment | Yes | Yes | No | Women were more frequently prescribed quetiapine and aripiprazole. |
| Cannavo, D., et al. | 2016 | Observational Study | Insight and recovery | Yes | Yes | No | Female sex predicts better insight and outcome. |
| Cao, H., et al. | 2021 | Observational Study | Adverse effects - QT prolongation | Yes | Yes | No | Women with SZ are more likely than men to exhibit QT prolongation on long-term antipsychotic therapy. |
| Centorrino, F., et al. | 2004 | Observational Study | Adverse effects - QT prolongation | Yes | Yes | No | Ziprasidone was more frequently discontinued in women than in men with SZ. |
| Ceskova, E., et al. | 2015 | RCT | Recovery after FEP | Yes | No | Yes | Olanzapine led to better PANSS outcome compared to other antipsychotics in women with SZ. |
| Charlotte, M., et al. | 2015 | Clinical Trial | Adverse effects | Yes | Yes | No | Women are prescribed less often antipsychotics with high risk for metabolic effects. |
| Chen, C. C. | 2024 | Clinical Trial | Genetic factors - C4 | Yes | No | No | Males and females with SCZ have different C4 haplotypes which may explain the later onset. |
| Chen, Y., et al. | 2023 | Comparative study | Neurobiology of SZ | Yes | No | No | Women have different transcription rates in numerous immune related genes linked to SZ compared to men. |
| Cheng, F., et al. | 2013 | Comparative study | Metabolic function | Yes | Yes | Yes | Male sex is a predictor for metabolic changes especially from olanzapine and clozapine. |
| Chung, Y. C., et al. | 2016 | Clinical Trial | Treatment of FEP | Yes | No | No | Females respond better to paliperidone in FEP. |
| Ciocca, G., et al. | 2015 | Observational Study | Adverse effects - sexual dysfunction | Yes | No | No | In females with FEP severity of symptomatology was linked to sexual dysfunction but not in males. |
| Costa, A. L. | 2024 | Observational Study | Adverse effects - oral hygiene | No | No | No | Men and women with SZ are equally likely to develop dental health problems. |
| Dama, M., et al. | 2019 | Observational study | Recovery after FEP | Yes | No | No | Women are more likely to achieve symptom remission. |
| Deligiannidis, K. M., et al. | 2013 | Clinical Trial | Symptomatology | Yes | Yes | No | Women exhibit more hallucinations than men in SZ. |
| Dittmann, R. W., et al. | 2010 | Clinical Trial | Adverse effects - depression | Yes | Yes | No | Women reported greater reduction of depression and somatic complaints. |
| During, S. W., et al. | 2019 | Observational study | Adverse effects - Hyperprolactinemia | Yes | Yes | No | Women reported greater sexual dysfunction and hyperprolactinemia. |
| Edinoff, A. N., et al. | 2021 | Review | Adverse effects - Hyperprolactinemia | Yes | Yes | No | Women with SZ exhibit hyperprolactinemia more frequently and may need to switch. |
| Efthimiou, O. | 2024 | Metanalysis | Adverse effects - Hyperprolactinemia | No | Yes | No | No difference found in sexual dysfunction. |
| Elliott, A., et al. | 2018 | Observational study | Adverse effects - QT prolongation | Yes | Yes | No | Females with SZ have clinically significant prolongation of QT under polypharmacy. |
| Ercis, M., et al. | 2024 | Review | Treatment response | Yes | Yes | No | Women with SZ have better outcomes than men but experience more adverse effects. |
| Fernando, P., et al. | 2020 | Review | Treatment response | Yes | No | No | Women and men with SZ need different guidelines for managing their symptomatology. |
| Friedrich, M. E., et al. | 2020 | Observational study | Adverse effects - QT prolongation | No | Yes | No | Ziprasidone and clozapine have the highest rates of QT prolongation and cardiac toxicity. |
| Fond, G., et al. | 2015 | Observational study | Treatment of toxoplasma in SZ | No | Yes | No | No sex differences found in treatment response to toxoplasma infection. |
| Frota, I. J., et al. | 2023 | Clinical Trial | Markers of inflammation | Yes | No | No | Women had higher inflammation markers, and higher dosage of antipsychotic correlated with worse functioning in women only. |
| Galbally, M., et al. | 2024 | Clinical trial | Antipsychotic efficacy | Yes | Yes | No | Risperidone only has a worse tolerability profile than other antipsychotics in males. |
| Gamon, V., et al. | 2021 | Observational study | Treatment patterns | Yes | Yes | No | Women were more likely to receive antipsychotic polypharmacy. |

**Supplementary Table 1** The table summarizes the main findings of all the reports found in our systematic review, categorizing them by study type, main focus, whether they uncovered a sex difference or not, whether a drug was used or not, whether a control group was used and the main finding if the study.
